# Structural and ultrastructural evidence for telocytes in prostate stroma

**DOI:** 10.1111/jcmm.12021

**Published:** 2013-02-07

**Authors:** Lara S Corradi, Mariana M Jesus, Ricardo A Fochi, Patricia S L Vilamaior, Luis A Justulin-, Rejane M Góes, Sérgio L Felisbino, Sebastião R Taboga

**Affiliations:** a Department of Biology, Institute of Biosciences, Humanities and Exact Sciences (IBILCE), Univ. Estadual Paulista – UNESPSão José do Rio Preto, São Paulo, Brazil; b Department of Morphology, Faculty of Pharmaceutical Sciences (FCF), Univ. Estadual Paulista – UNESPAraraquara, São Paulo, Brazil; c Department of Morphology, Institute of Biology (IB), Univ. Estadual Paulista – UNESPBotucatu, São Paulo, Brazil

**Keywords:** Telocyte, ventral prostate, stromal cell, CD 34

## Abstract

The prostate comprises a glandular epithelium embedded within a fibromuscular stroma. The stroma is a complex arrangement of cells and extracellular matrix (ECM) components in addition to growth factors, regulatory molecules, remodelling enzymes, blood vessels, nerves and immune cells. The principal sources of ECM components are fibroblasts and smooth muscle cells (SMC), which synthesize the structural and regulatory components of the ECM. Telocytes (TCs) were recently described as a novel stromal cell type that exhibited characteristic features. The aim of this study was to confirm the presence of TCs in prostate stromal tissue of gerbils, as the stromal compartment of this gland is a dynamic microenvironment. We used transmission electron microscopy (TEM), light microscopy and immunohistochemistry methods to provide morphological evidence for the presence of TCs. Cells that resembled TCs were observed in gerbil prostatic stroma. These cells had small cellular bodies with very thin and extremely long cellular processes. They were found primarily in the subepithelial area and also at the periphery of SMC layers. TCs also exhibited moniliform processes, caveolae and nuclei surrounded by small amounts of cytoplasm. Close contacts between TC podomers were evident, particularly in the adjacent epithelial compartment. This morphological evidence supported the presence of TCs in the gerbil prostatic stroma, which we report for the first time.

## Introduction

The prostate is an important accessory gland that is located in the mammalian genital tract. In conjunction with seminal vesicles, it produces the bulk of seminal fluid [1]. This gland comprises a glandular epithelium embedded within a fibromuscular stroma. The stromal compartment is a complex arrangement of stromal cells and extracellular matrix (ECM) components in addition to growth factors, regulatory molecules, remodelling enzymes, blood vessels, nerves and immune cells. These components act in a coordinated manner to regulate cell function and maintain overall prostatic tissue homeostasis [2, 37].

The principal sources of ECM components are fibroblasts and smooth muscle cells (SMC), which synthesize the structural and regulatory components of the ECM [2, 3]. The growth and development of the prostate depend on the levels of circulating androgens produced by the testes. Its homeostatic state during adult life is maintained by these steroid hormones, which act *via* stromal–epithelial interactions [4, 5]. Thus, in adulthood, a balance between cell proliferation and cell death is established so that no further growth occurs in the prostate gland [6].

However, during ageing in humans and several other species, including dogs and some rodent strains, cellular hyperplasia may occur despite a decrease in the production of sexual hormones, such as testosterone, which results in age-dependent prostatic hyperplasias [7]. These alterations may evolve into prostate cancer, a disease that affects men worldwide. Prostate cancer results from a lesion whose heterogeneous behaviour is still not completely understood. Thus, there is significant interest in the morphology, components and behaviours of the prostate during its normal development and in different disorders.

Studies of the human prostate, as well as of breast and colon cancer specimens, have identified stromal cells that are phenotypicaly altered cells, primarily fibroblasts and SMCs, in addition to modified ECM compositions, including new formation and deposition of abundant fibrillar collagens and increased capillary density [2, 8]. Corradi *et al*. [9, 10] noted general stromal rearrangements in the prostate glands of young, adult and old gerbils (*Meriones unguiculatus*) after blocking steroidogenic enzymes. These changes included the accumulation of fibrillar components and phenotypically altered SMCs and fibroblasts. However, detailed, specific information on these cells' morphologies and immunophenotypes has not been acquired.

A novel stromal cell type was recently described. Popescu *et al*. [11, 12] noted that interstitial Cajal-like cells (ICLCs) were obviously not similar to the canonical interstitial cells of Cajal (ICC). Thus, they proposed the name Telocytes (TCs) to describe this novel cell type. In general, TCs have small cell bodies and exhibit variable numbers of long, thin cellular elongations designated telopodes (Tp). Since then, research on the presence and function of TCs has increased exponentially and provided relevant information to the study of this new cell type [11–13].

Various *in vitro* (isolated cells in culture) and *in vivo* (fixed specimens) techniques and light, fluorescent, transmission and scanning electron microscopic have been used to characterize these cells morphologically [13, 14]. Also, immunohistochemistry is of fundamental importance to determine the phenotype(s) of TCs, in addition to evaluating their pathological changes. Bucharest's group tested numerous antibodies to find a single marker that could be considered specific for TCs [15–17]. However, TCs might have different immunohistochemistry profiles among different organs and even within the same organ [13]. TCs have already been identified in the stroma of several organs, such as heart [11, 17–19], intestine [20], gallbladder [21], uterus and fallopian tube [22–24], mesentery [25], pulmonary veins [26], trachea and lungs [14, 27, 28], pleura [29], placenta [30], skeletal muscle [31], exocrine pancreas [32], skin [33], mammary gland [34] and parotid glands [35].

The identification of TCs, which have remarkably long, thin and moniliform processes, is based primarily on the recognition of Tp's [11]. These Tp's have a number of distinctive characteristics, including their length, thinness, moniliform processes and a branching network pattern, which forms a labyrinthine system by 3D convolution and overlapping and communications through junctions [12, 13]. Identifying these characteristics by ultrastructural analysis is essential. Under higher magnification, the moniliform processes are characterized by alternating thin fibrillar-like segments (podomers) and dilated, cisternae-like regions (podoms) and the presence of caveolae, an endoplasmic reticulum, and mitochondria [35]. To date, it has been found that TCs are positive for c-kit (CD 117), CD 34 and vimentin [11] by immunohistochemistry.

The aim of this study was to confirm the presence of TCs in the prostatic stroma of gerbils, used as a rodent experimental model [7, 9, 10]. To provide morphological evidence for TCs, we used both Transmission Electron Microscopy (TEM) and light microscopy. The morphological evidence and immunohistochemistry assay results presented in this study appear to confirm the presence of this novel cell type, TCs, in the gerbil prostatic stroma. To our knowledge, this is the first report of these cells in prostate stroma.

## Materials and methods

### Animal protocols

Thirty adult male gerbils (*Meriones unguiculatus*, Gerbilinae, Criscetidae) were housed under controlled conditions of temperature (25°C), relative humidity (40–70%) and light (12-hr light-dark cycle), and were allowed free access to standard chow and water. Gerbils were first weighed (≍80.0 gm), anaesthetized by CO_2_ inhalation and decapitated. The prostatic complex was dissected out, weighed and fixed according to the different protocols described below. The ventral prostate was carefully dissected out, weighed and fixed. Animal handling and experiments were in accordance with the guidelines of the Ethics Commission for Animal Experimentation (CEEA) of Campinas State University – UNICAMP, São Paulo, Brazil (Process no.: 1236-1), which followed the Guide for Care and Use of Laboratory Animals.

### Structural analysis

The entire prostate was dissected out and weighed. Only the ventral lobe was fixed by immersion in Karnovsky solution (4% parformaldehyde, 2.5% glutaraldehyde in 0.1 M phosphate buffer, pH 7.2) for 24 hrs or immediately frozen in liquid nitrogen for later cryosectioning. After fixation, tissues were washed with running tap water, dehydrated in a graded ethanol series and embedded in glycol methacrylate resin (Leica Historesin Embedding Kit; Leica, Nussloch, Germany). Some prostatic fragments were fixed in 4% formaldehyde freshly prepared in phosphate buffer (pH 7.2) for 24 hrs. These were also dehydrated in a graded ethanol series and embedded in Paraplast (Histosec-Merk, Darmstadt, Germany). Tissue sections (3–4 μm) were obtained using a Leica automatic rotary microtome (Leica RM2155; Leica). For general studies, histological sections were stained with haematoxylin and eosin [36]. Microscopic analyses used a Zeiss-Jenaval (Zeiss-Jenaval, Jena, Germany) or an Olympus BX60 light photomicroscope (Olympus, Hamburg, Germany). Microscopic fields were digitized using Image-Pro Plus version 4.5 for Windows™ software (Media Cybernetics Inc., Bethesda, MD, USA).

### Ultrastructural analysis

Prostate ventral lobe fragments were minced into small pieces and fixed by immersion in 3% glutaraldehyde plus 0.25% tannic acid solution in Millonig's buffer, pH 7.3, containing 0.54% glucose for 24 hrs [9]. After washing with the same buffer, samples were post-fixed with 1% osmium tetroxide for 1 hr, washed in buffer, dehydrated in a graded acetone series and embedded in Araldite resin. Ultrathin sections (50–75 nm) were prepared using a diamond knife and stained with 2% alcoholic uranyl acetate for 30 min. followed by 2% lead citrate in a 1 M sodium hydroxide solution for 10 min. Samples were evaluated by electron microscopy using a LEO – Zeiss 906 TEM at 80 kV.

### Digital colouring of TEM images

To provide evidence for TCs among several stromal structures of the gerbil prostate, we digitally coloured these cells on electron photomicrographs to make TCs more visible. All elements were carefully hand coloured using Adobe Photoshop software (Adobe Systems, Adobe Acrobat 9 Pro Extended. Inc., New York, NY, USA).

### Immunohistochemical analysis

We used 5-μm-thick cryosections for immunohistochemical analysis. Endogenous peroxidase activity was blocked using 0.3% H_2_O_2_ in methanol for 30 min. Then, sections were washed in PBS and incubated with 3% BSA for 1 hr. Incubation with a primary antibody was at 4°C overnight, followed by incubation with a peroxidase-labelled polymer (mouse on rat HRP polymer, BioCare Medical, ref. MRT621 H) for 60 min. and incubation with a substrate chromogen (3,3′ diaminobenzidine tetrahydorchloride) for 15 min. Between each step, sections were thoroughly rinsed three times with phosphate buffered saline, pH 7.2. Counterstaining for nuclei was with haematoxylin for 10 sec. The primary antibody used for TCs was anti-CD34 (mouse CD 34, clone sc-74499, Lot # A1810, Santa Cruz Biotechnology, 1/100; Santa Cruz Biotecnology Inc., Santa Cruz, CA, USA). Negative controls omitted the primary antibody. Images were acquired using an Olympus BX 60 microscope equipped with an Olympus digital camera (Olympus).

## Results

### Light microscopy and immunohistochemistry results

**Fig. 1:**
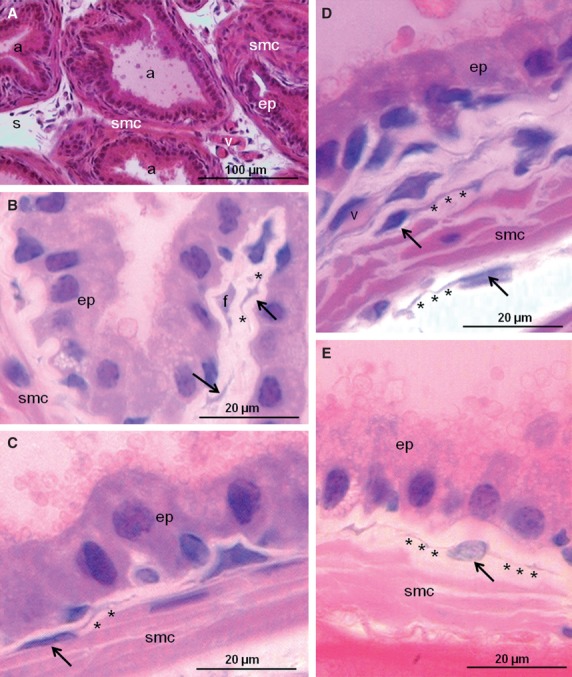
Prostate haematoxylin and eosin stained sections. (**A**) General features of the gerbil ventral prostate exhibiting acini (a) and periacinar smooth muscle cells (smc) organized in a densely packed layer, which is surrounded by connective tissue stroma (s). The epithelium (ep) is tall columnar. Blood vessels (v). (**B**–**E**) Telocytes (arrows) can be observed adjacent to the epithelial (ep) compartment and at the periphery of the smc layer. The phenotypic characteristics of this novel cell type are shown by the small cellular body with the presence of telopodes (asterisks) that are very thin and extremely long. Fibroblast (f).

As shown in Figure 1A, for the acini of the gerbil ventral prostate, the glandular epithelial compartment was surrounded by vascularized connective tissue stroma in which smooth muscle bundles surrounded each acinus. The epithelium was simple or pseudostratified and comprised columnar cells. In the stroma, a complex arrangement of cells and ECM components, SMCs were well organized in a densely packed layer surrounding the acinar epithelium. Moreover, collagen and reticular fibres were noted that were interspersed throughout the stroma, primarily along the basal aspect of epithelial structures, but also among SMCs. Immune cells, fibroblasts, blood vessels and nerves were also found in the stromal compartment.

**Fig. 2:**
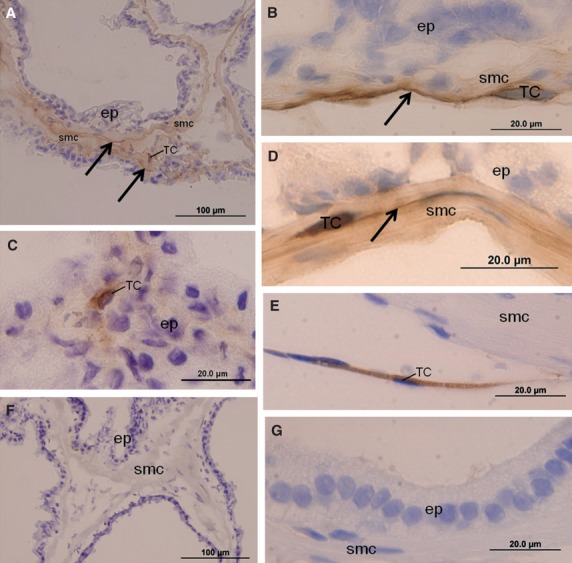
Immunohistochemistry assay for CD34 in prostate cryosections. (**A**) General features of tissue immunoreactivity for CD34 in telocytes (TC) and its cellular elongations (arrows). (**B**–**E**) Details for isolated images of a TC and its elongations (arrows). (**F**–**G**) Negative control. smc (smooth muscle cell), ep (epithelium), TC (telocytes).

**Fig. 3:**
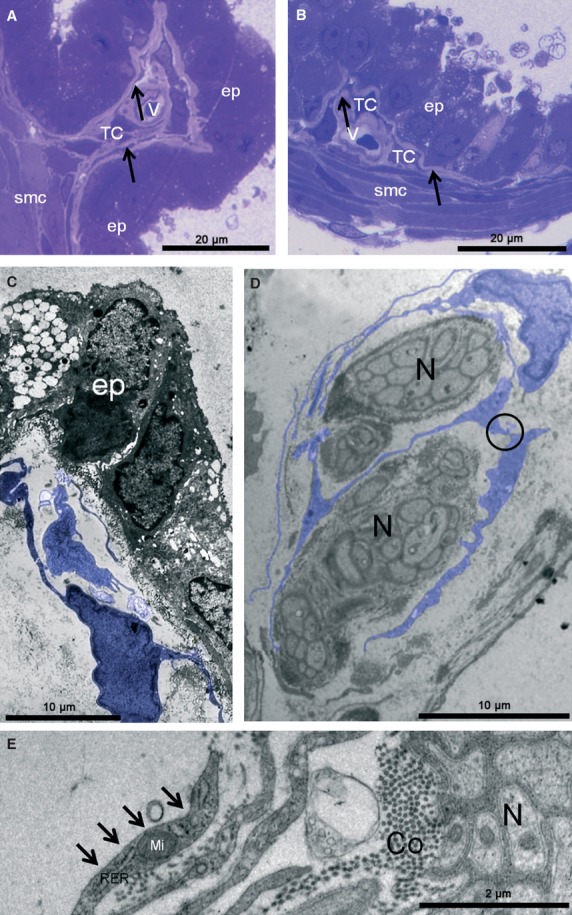
Prostate semi-thin sections for light microscopy and ultra-thin sections for transmission electron microscopy. (**A** and **B**) Detailed view of a telocyte (TC) and its elongations (arrows) in the subepithelial region. These images were obtained with light microscopy for 0.5-μm sections. (**C**–**E**) Ultrastructural features of prostate telocytes (TC). The distribution of this cell type was verified primarily in the subepithelial region. The TC is identified in these figures in blue (C and D). (D) Two telocytes close to prostatic nerves (N) showing extremely thin, long processes extending from the cellular body, with repeated curving. Black circle indicates a close contact between two TCs. (E) Detail of TC located in the perineural region. Note that the extensions of the TC are not observed basal lamina (arrows). ep (epithelium), smc (smooth muscle cell), v (blood vessel), RER (rough endoplasmic reticulum), Mi (mitochondria), TC (telocyte), N (nerve), Co (collagen).

In the area adjacent to the epithelium, TCs were readily identified as small bodies with thin, long processes that were characteristic of the structures of Tp's. Also, the same types of cells were noted at the periphery of the SMC layer. The small body of TCs could assume a piriform, spindle or triangular shape and Tp's that extended from the body were thin (Figs 1B–E, 3A and B). Immunohistochemical analysis showed that the prostatic stroma had cells that were strongly positive for staining with an anti-CD 34 antibody and had cytoplasmic elongations that corresponded to those described for TCs (Fig. 2).

### Transmission electron microscopy results

**Fig. 4:**
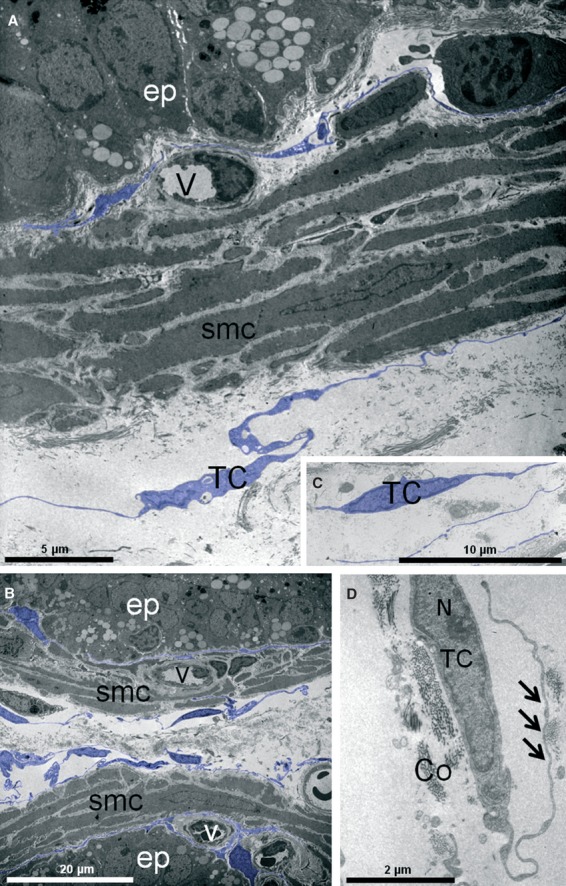
Transmission electron microscopy of prostate. (**A**) Telocytes (digitally coloured in blue) are noted adjacent to the epithelial (ep) cells and in the periphery of the smooth muscle (smc) layer. In this region, the moniliform feature of a Telopodes (digitally coloured in blue) can be observed, along with podomers (thin fibrillar-like segments) and podoms (dilated, cisternae-like regions). (**B**) In the stromal region between two acini, next to smc, numerous TCs were observed that exhibited very thin, long processes. (**C**) Phenotypic profile assessments of a TC showing a small, spindle-shaped body with a small amount of cytoplasm and thin, long processes in the prostate stroma. (**D**) General aspect of the isolated stromal TC surrounded by collagen fibrils (Co). This cell exhibits a thin podomer (arrows).

**Fig. 5:**
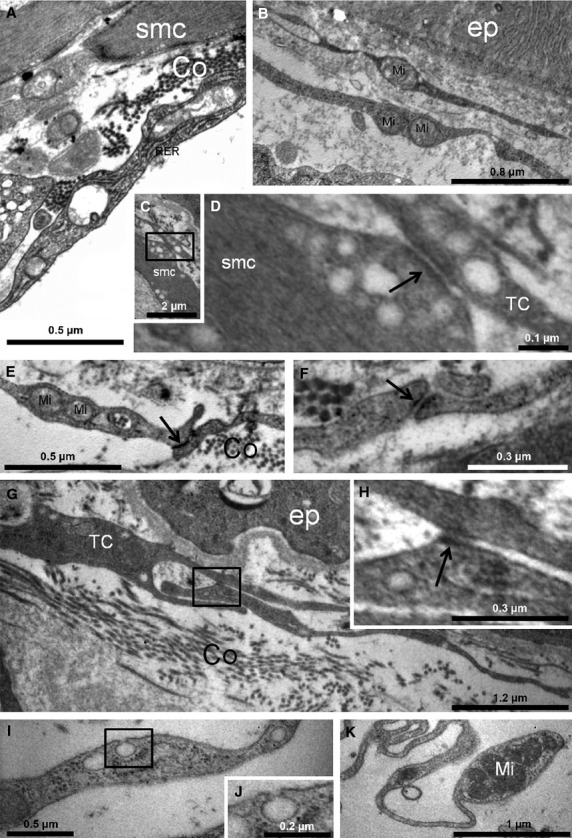
Transmission electron microscopy. High-magnification images of podomers (**A** and **G**), podons (**F** and **K**), “adherens junctions” (arrows) (**C**, detail in **D**, F and **H**), gap junction (arrow) (**E**), moniliform elongations containing mitochondria (**B** and E) and caveolae (**I** and **J**). smc (smooth muscle cell), ep (epithelium), RER (rough endoplasmic reticulum), TC (telocyte), Mi (mitochondria), Co (collagen fibrils).

Electron microscopy analysis confirmed the presence of cells having the ultrastructural features of TCs in the gerbil prostatic stroma (Figs 3C–E, 4A–D and 5A–K). These cells were mostly found adjacent to prostatic epithelium, although some were interspersed in the SMC layer and at the SMC layer periphery (Figs 3C, 4A and B). These results were in accordance with those obtained with light microscopy.

The major characteristics of the TC cell body are scarce cytoplasm around the nucleus and its spindle shape. The ultrastructural TC pattern in the stroma of the gerbil prostate exhibited a small, spindle-shaped body and two to three thin, very long Tp's, with some that were in close contact similar to adherens junctions (Fig. 5C–H). In addition, several multiple contact connections and/or ‘adherens junctions’ between TCs were observed among these cells in the prostatic stroma (Figs 3D and 5D–H).

Contacts between Tp's were found, especially among TCs in the subepithelial area. Regular moniliform processes, with alternating thin segments (podomeres; Fig. 4C) and dilations (podoms), which were rich in mitochondria (Fig. 5B, E and K), endoplasmic reticulum (Figs 3E and 5A) and caveolae (Fig. 5I and J) were also noted. Mitochondria were typically noted in the dilation processes and the endoplasmic reticulum was located primarily in the body region (Fig. 5B). Some TCs that were observed close to prostatic nerves had extremely thin, long processes that extended the cellular body with repeated curving (Fig. 3D and E).

## Discussion

A number of animal models, particularly mice, have been used for research on prostate biology and prostate diseases [38]. These studies have found both significant similarities and differences in rat, mouse and human prostate glands and have analysed the potential of these models to recapitulate human prostatic disease [36, 39]. The rodent prostate complex comprises four distinct paired lobes, ventral, lateral, dorsal and anterior, each of which serves a particular function with regard to histology and secretory protein production [40]. The morphology of the human prostate is more compact, without distinct lobes, and is divided into three zones: central, transitional and peripheral [41].

The Mongolian Gerbil is a small rodent of the Muridae family, subfamily Gerbillinae, species *Meriones unguiculatus*, which has become increasingly used as an experimental model for studies on male and female prostate histophysiology, as the female of this species has a functionally active prostate gland [42]. Unlike the prostate glands of mice and rats, the gerbil's prostate morphology is similar to that of the human gland with respect to the fusion of its lobes in a compact structure [7]. The ventral gerbil prostate has been evaluated morphologically and quantitatively throughout postnatal development. Previous results from our laboratory demonstrated that the histological, histochemical and ultrastructural features of the adult gerbil's prostate were comparable with those of the human prostate [7, 9, 10, 42, 43].

Little research has been done on the prostatic stroma that forms a dynamic microenvironment, which is fundamentally important for prostate growth and development during foetal life and for homeostasis in adulthood and within which compartment TCs appear to be present [44]. The suspected presence of this novel stromal cell type [11] was first noted in the gerbil prostate by light microscopy when a cell with a different morphology from those of typical fibroblasts was observed in the subepithelial region. The newly reported TC's were described as having peculiar features, with very long, thin and moniliform processes called Tp's. Identification of this cell was mostly based on the recognition of Tp's [11].

These very long, thin processes could be clearly observed in the gerbil prostatic stroma, especially in the adjacent epithelial area, in addition to the periphery of SMC layers. This suggested the hypothesis that these cells were not fibroblasts. However, to confirm the moniliform feature, in addition to a branching network pattern and communication through junctions between TCs, a higher magnification view with TEM is also required [32].

Ultrastructurally, we confirmed the presence of TCs in the prostate stroma of the adult gerbil. To our knowledge, this is the first report of TCs in prostatic stroma. This was based on finding moniliform processes with podomers (thin fibrillar-like segments) and podoms (dilated, cisternae-like regions). In addition, these regions were rich in caveolae, endoplasmic reticulum and mitochondria, as observed for TCs in heart tissues [12, 16] and in parotid glands [32].

Immunohistochemistry has been performed for the gerbil prostate and TCs might show different immunohistochemical profiles among organs and even in the same organ. Bucharest's group tested numerous antibodies in an effort to find a single marker that could be considered specific for this cell type or, at least, specific for the TCs of a given organ. Determining the phenotype(s) of TCs is of fundamental importance, as this would provide for their unequivocal identification and would also aid in evaluating their size, shape, number and, ultimately, their movements, migration and pathological changes [13].

C-kit-positive interstitial Cajal cells have been demonstrated in the human prostate [45], and a vimentin-positive population of interstitial cells has been demonstrated in the dog prostate [46]. Our results show for the first time that there are CD 34-reactive cells in the prostate stroma, which reinforces the presence of these TCs in prostate stromal tissue.

Several roles have been suggested for TCs, most of which are plausible and not mutually exclusive. However, to date, none of these have been totally proven. As reported by Popescu and Faussone-Pellegrini [11], TCs might provide mechanical support by guiding the migration of other cells to define the final organization of an organ or its repair or renewal. Intriguingly, a role in neurotransmission in the gut has also been suggested, which possibly contributes to spreading the slow waves generated by interstitial Cajal cells. TCs might also be involved in intercellular signalling.

Cardiac TCs have been suggested as having a nursing role, and those in the oviduct and myometrium are suggested to be sensors of steroid hormones [13]. For the prostate, TC functions and pathology have been suggested, including involvement in benign prostatic hyperplasia and cancer, although further studies are required. While it is too early to propose roles for gerbil prostate TCs, evidence of their involvement in maintaining prostate tissue architecture is justified based on their locations.

The TCs that appear in the subepithelial region along with those observed at the periphery of smooth muscle might ensure the homeostasis of the stromal compartment for generating the compartmentalization of prostatic tissue microenvironments. Studies of the lower urinary tract demonstrated that the guinea pig prostate contained a network of c-kit-positive interstitial cells that was between the glandular and stromal muscle layers of individual prostatic acini as well as between individual SMCs of the stroma [47, 12]. These authors' discovery of slow waves and interstitial Cajal cells in the guinea pig prostate has prompted some interest in the prostate literature [45], as it suggests that slow-wave activity might be responsible for the spontaneous tonus of the SMC in the human prostate. Thus, this might be TCs function in the gerbil prostate, as their localization similar to that proposed by Lang and Klemm [47] is strategic for spontaneous contraction.

Telocytes might be novel target cells for the prevention and treatment of disease. This novel cell type within the prostatic microenvironment has become an important tool for our research group, as it provides insights into numerous unanswered questions regarding the biology of the prostate and the effects of endocrine disrupting chemicals on the prostatic stroma. The functions and pathology involving TCs in the prostate will require further studies, including studies on benign prostatic hyperplasia and cancer. Investigating the biological functions of TCs within the prostate is important, as the stromal compartment plays key roles in both normal and abnormal tissues. The presence of TCs in the stroma of the gerbil prostate provides a new tool for understanding responses that have not yet been determined in prostate biology. The purpose of this study was primarily to confirm the presence of a novel cell type, TCs, in the prostate based on the specifics of their ultrastructural and immunohistochemical characteristics.
